# Effects of acute wearable resistance loading on overground running lower body kinematics

**DOI:** 10.1371/journal.pone.0244361

**Published:** 2020-12-28

**Authors:** Karl M. Trounson, Aglaja Busch, Neil French Collier, Sam Robertson

**Affiliations:** 1 Institute for Health and Sport, Victoria University, Footscray, Victoria, Australia; 2 Western Bulldogs Football Club, Footscray, Victoria, Australia; 3 University Outpatient Clinic, Sports Medicine & Sports Orthopedics, University of Potsdam, Potsdam, Germany; University of Rome, ITALY

## Abstract

Field-based sports require athletes to run sub-maximally over significant distances, often while contending with dynamic perturbations to preferred coordination patterns. The ability to adapt movement to maintain performance under such perturbations appears to be trainable through exposure to task variability, which encourages movement variability. The aim of the present study was to investigate the extent to which various wearable resistance loading magnitudes alter coordination and induce movement variability during running. To investigate this, 14 participants (three female and 11 male) performed 10 sub-maximal velocity shuttle runs with either no weight, 1%, 3%, or 5% of body weight attached to the lower limbs. Sagittal plane lower limb joint kinematics from one complete stride cycle in each run were assessed using functional data analysis techniques, both across the participant group and within-individuals. At the group-level, decreases in ankle plantarflexion following toe-off were evident in the 3% and 5% conditions, while increased knee flexion occurred during weight acceptance in the 5% condition compared with unloaded running. At the individual-level, between-run joint angle profiles varied, with six participants exhibiting increased joint angle variability in one or more loading conditions compared with unloaded running. Loading of 5% decreased between-run ankle joint variability among two individuals, likely in accordance with the need to manage increased system load or the novelty of the task. In terms of joint coordination, the most considerable alterations to coordination occurred in the 5% loading condition at the hip-knee joint pair, however, only a minority of participants exhibited this tendency. Coaches should prescribe wearable resistance individually to perturb preferred coordination patterns and encourage movement variability without loading to the extent that movement options become limited.

## Introduction

Across many field-based sports, athletes must be capable of running long distances throughout a match [1–3]. Depending on the sport, total running distance can range from an average of 6 km in rugby league to 12 km in Australian Rules football [1]. In Australian Rules football, soccer, rugby league, and rugby sevens, most of the distance covered during match play can be classified as “low-intensity activity”, i.e., occurring at velocities <5.4 m.s^-1^ [1, 3]. While high-intensity efforts are often associated with significant match events, adequate sub-maximal running capabilities are also important for effective opponent tracking and retention of team formations during different phases of play throughout a match [4]. As such, training aimed at developing sub-maximal overground running performance is evidently worthwhile.

Development of sub-maximal running performance for field-based athletes is a multifactorial proposition and requires training of aerobic capacity, biomechanical factors for superior economy, and muscular strength [5–8]. Coaches should address these factors in training prescription and, in addition, athletes’ ability to adapt their running coordination patterns in accordance with the dynamic constraints of the sport [9, 10]. The capacity to exhibit “adaptability” in this sense allows for greater maintenance of performance in varied contexts and is a hallmark of higher performing athletes in many sports [10–13]. In field-based sport, organismic constraints in the form of local metabolite accumulation from intermittent anaerobic efforts [14, 15], muscle damage arising from high force eccentric contractions during decelerations [16], and muscular contusion from compressive force impacts [17], all present scenarios in which there is a challenge to an athlete’s preferred running coordinative structure, which must be adapted to.

Critically, the implementation of a training intervention aimed at encouraging movement variability in diving [10] suggests that the capacity for athletes to harness movement system degeneracy to maintain a performance outcome is trainable. This notion is further supported by nonlinear pedagogical training interventions in youth tennis [18]. Individuals exposed to greater task variability during training displayed a greater number of unique movement clusters, indicating the presence of degeneracy, during performance tasks. Exposure to task variability drives exploration of alternate movement strategies, or movement variability, as movement is adjusted to satisfy novel task demands [19]. Training in this way affords individuals the ability to adapt movement to maintain task performance under the varied constraints occurring in the dynamic sporting environment [10, 18, 20].

In the context of sub-maximal running kinematics, the effects of deliberately induced task variability through perturbation have been explored in research using elastic tubes attached from the hips to the ankles [21–23]. This intervention increases joint kinematic variability acutely, after which there is relatively rapid stabilisation around a slightly shifted coordinative structure [21, 23]. Although no post-training running test under novel conditions was undertaken, the performance benefits associated with exposure to constraints, which encourage movement variability in this way, are widely reported [24–28].

It is also worth noting that analyses of kinematic variability induced by constraint implementation to date have typically focussed on group-level changes [21, 29, 30]. Increasingly, there is support for individual-level consideration given that intrinsic behavioural dynamics and baseline kinematic characteristics alter the extent to which a particular constraint is experienced as a perturbation to the system [31–33]. Kinematic changes may vary markedly between individuals, which may not be clear when considering generalised responses, yet is important in a practical setting [34–36].

Lightweight wearable resistance (WR) may be a useful training tool for encouraging exploration of movement system degeneracy through movement variability. WR involves attachment of small weights to particular body segments, such as the trunk, arms, thighs, and shanks [37]. To date, research has considered WR in its capacity as a movement specific overload stimulus [38, 39], however, WR also presents a perturbation to coordination, which may induce movement variability. WR application alters segment inertial properties and as such can be considered an organismic constraint [40, 41]. Exposure to WR may ultimately be a useful stimulus for developing adaptable movement behaviours among athletes in preparation for changing organismic constraints faced during match play. This study aimed to describe the extent to which different acute lower limb WR loadings (1%, 3%, and 5% of body weight) alter coordination and induce movement variability during sub-maximal overground running. By considering both group- and individual-level responses, findings will provide context for coaches seeking to promote movement variability without imposing an excessive perturbation that limits movement options.

## Materials and methods

### Participants

Fourteen participants (three female and 11 male; mean ± SD: age 28.3 ± 4.4 years; height: 179.9 ± 7.6 cm; body mass: 76.8 ± 6.1 kg) volunteered to participate in this study. Participants were included on the basis that they were currently undertaking, or had recent previous experience (past year), in structured field-based sport competition. Participants in the study had no prior experience with WR. All participants provided written informed consent and were free from injury at the time of testing. All procedures used in this study complied with the criteria of the declaration of Helsinki and the ethical approval granted by the Victoria University Human Research Ethics Committee.

### Procedure

#### Data collection apparatus

**Fig 1:**
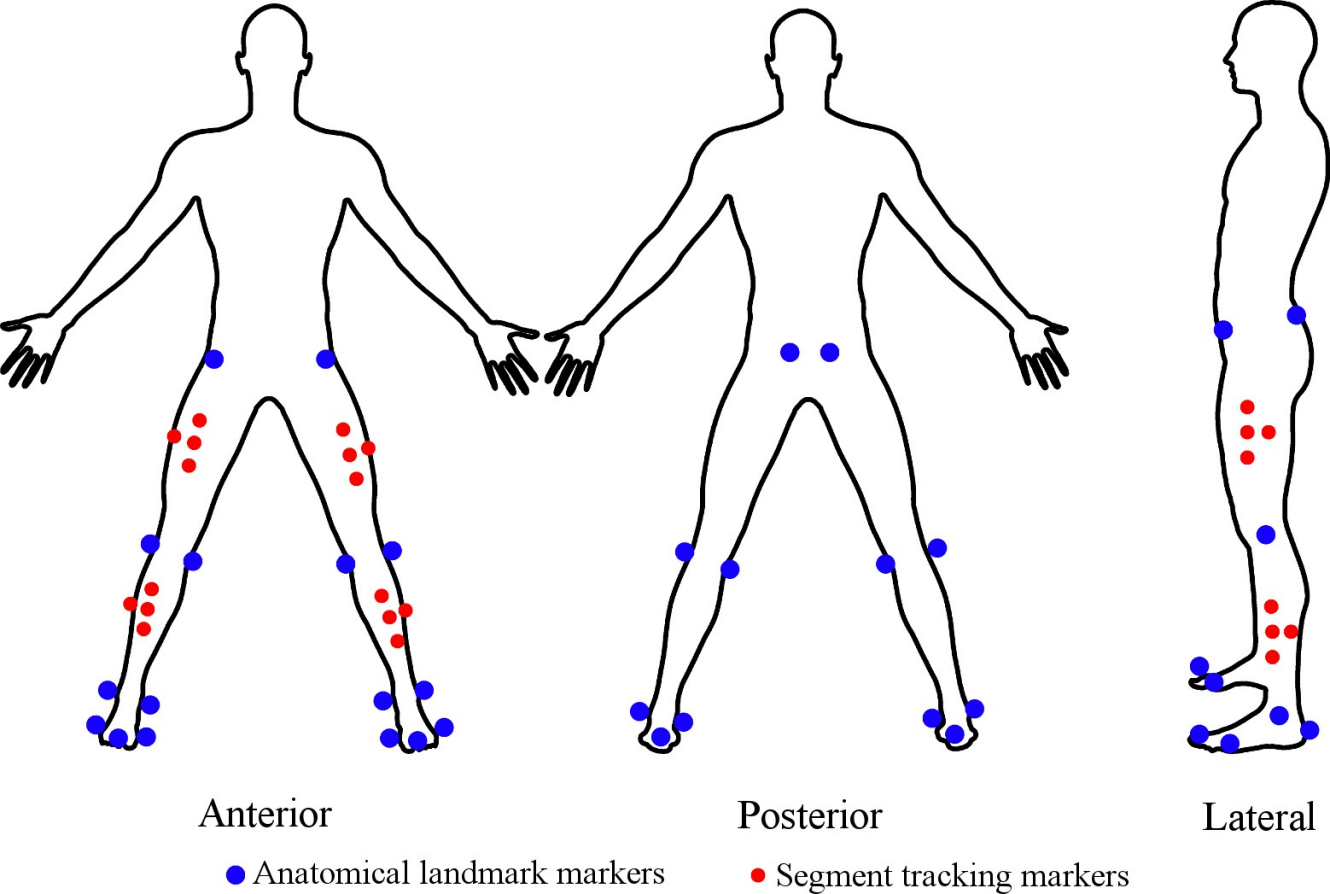
Lower body Plug-In-Gait model. Blue markers define the required anatomical landmarks, red markers are used for tracking segments.

A 10-camera VICON motion analysis system (T-40 series, Vicon Nexus v2, Oxford, UK) sampling at 250 Hz was used for collection of kinematic data. A total of thirty-six reflective markers with 14mm diameter were attached to lower body landmarks on the pelvis, thighs, shanks, and feet according to the Plug-In-Gait model (Plug-In-Gait Marker Set, Vicon Peak, Oxford, UK) (Fig 1).

#### Wearable resistance

**Fig 2:**
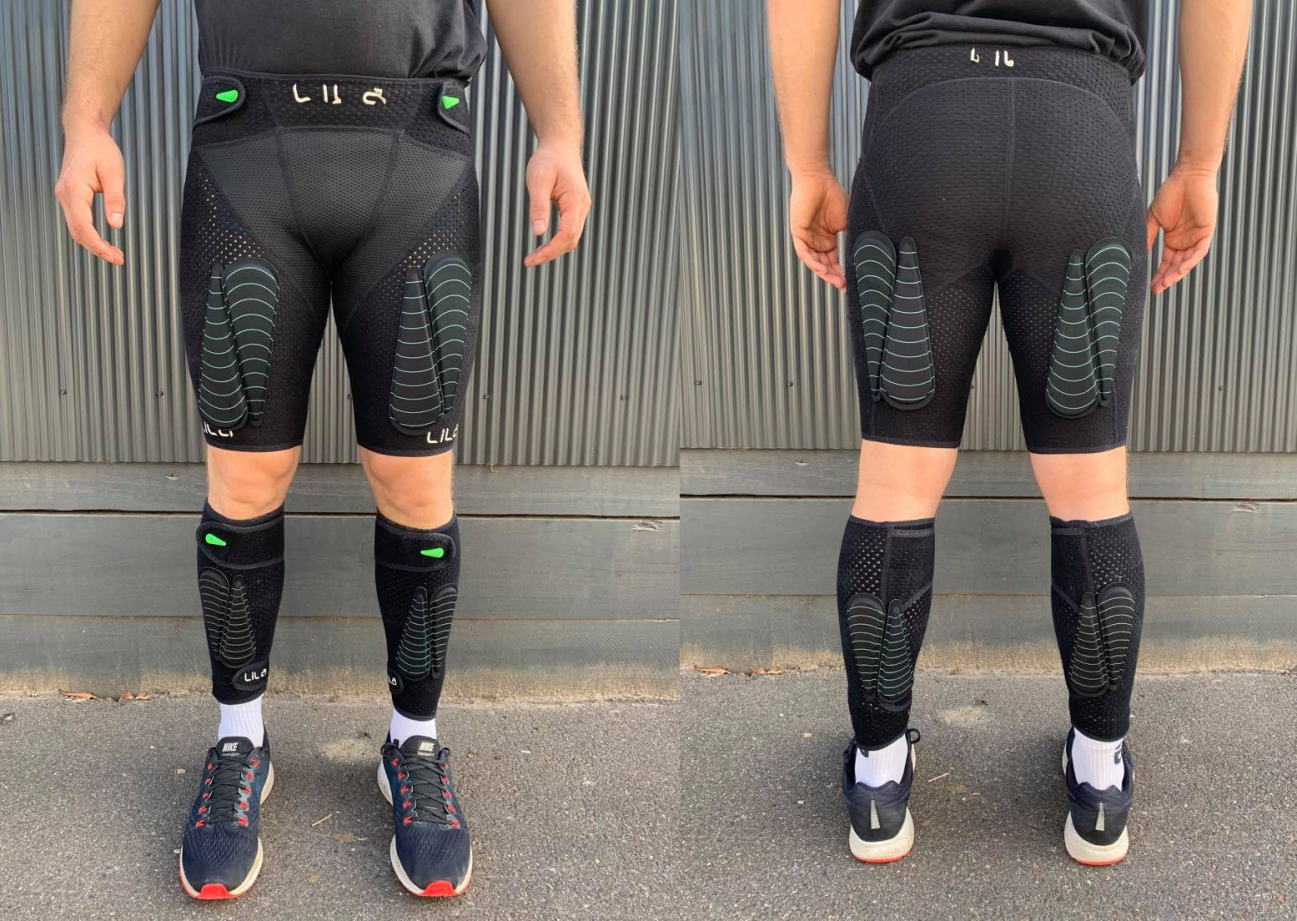
Lila™ Exogen™ compression shorts and calf sleeves with thigh and shank loading.

Throughout testing, participants wore Lila^TM^ Exogen^TM^ (Sportboleh Sdh Bhd, Kuala Lumpur, Malaysia) compression shorts and calf sleeves. During WR exposure trials, a combination of 50, 100, and 200g fusiform shaped loads (with Velcro backing) totalling the required proportion of participants’ body weights were attached to the compression garments (Fig 2). Loads were distributed in a 2:1 thigh:shank ratio about the centre of mass of each segment [42]. The required loads were added in an alternating fashion between the anterior and posterior surfaces, and between a proximal-dominant and distal-dominant orientation, in order to avoid a large shift in the centre of mass of each segment.

#### Experimental setup

Testing was undertaken on a 20 m section of the Biomechanics Laboratory at Victoria University. Motion analysis cameras were arranged around the 10 m mark of the 20 m section and the approximate capture volume was 6.0 m long, 2.5 m high, and 3.0 m, wide.

#### Data collection

Following application of compression garments and attachment of reflective markers, participants undertook an initial warm-up in which they ran back and forth along the 20 m section in a “shuttle” fashion for 2 min. Running velocity was dictated through the use of an audible metronome, which counted each second from 1–9, before repeating for every subsequent shuttle. Participants underwent a 2 min rest period following the first warm-up run before performing a second warm-up run for 1 min at an increased velocity defined by 6 s shuttle efforts. Owing to the requirement of 180° changes of direction after each shuttle, running velocities achieved through the capture area were greater than the theoretical straight-line velocity of 3.3 m.s^-1^. Analysis of pilot data showed mean ± SD velocities of 4.16 ± 0.36 m.s^-1^ through the capture area. Such velocities are commonly described as “striding” or “running” in field based sports, but fall below the “high-intensity” classification, often defined as >5.4 m.s^-1^ [43, 44]. The first trial was performed with body weight only (BW), and participants completed 2 min worth of 20 m shuttles with the 6 s pacing speed per shuttle. Captures were taken each time participants passed through the 10 m mark capture area during runs from the start point to the 20 m mark only. Captures were not performed on the return shuttles. This process yielded capture of 10 complete strides across the 2 min trial.

Participants performed three subsequent 2 min running trials in which they were allocated WR loading of 1%, 3%, and 5% of body weight in a randomised order. Each trial was interspersed with a 3 min rest period. The result of this protocol was 10 complete overground running strides per condition, per participant.

### Data processing

Visual 3D software (C-motion, Rockville, MD, USA) was used to construct a four segment model (pelvis, thigh, shank, and foot) for each participant. Within each participant, the leg on which most complete strides were successfully captured was used for analysis. This approach maximised available data given that individual stride characteristics tended to allow one side to be captured more consistently within the bounds of the 6 m capture area (see S1 Table for the leg used for each participant). Runs in which several marker trajectories were lost or accurate model construction could not be satisfied were excluded from analysis. Out of a possible 560 runs per-joint, 510 were successfully reconstructed for the hip, 530 for the knee, and 521 for the ankle. For a record of excluded runs and the participants and runs to which these pertained, see S1 Table. For successfully reconstructed runs, marker trajectories were smoothed via a fourth order low-pass Butterworth filter with 10 Hz cut-off frequency, based on mean residual amplitudes [45]. Each run was trimmed to one complete stride cycle, which was defined as the period between two consecutive toe-off events on the same limb. Toe-off was defined by the initial rise in vertical displacement of the toe marker proceeding its lowest point at the end of the support phase [46, 47]. Time-continuous sagittal plane joint angles for the hip, knee, and ankle (^o^) were normalised to 100% of the stride cycle for further analysis. Positive and negative joint angles were defined relative to the positions of joints in upright standing. Positive joint angles indicate positions of hip flexion, knee flexion, and ankle dorsiflexion relative to standing, while negative joint angles indicate positions of hip extension, knee extension, and ankle plantarflexion relative to standing.

### Data analysis

Running velocity was compared across different loading conditions using a one-way repeated measures ANOVA with Bonferroni correction applied to post-hoc pairwise comparisons. A significance level of *α* = 0.05 was used.

#### Statistical parametric mapping t-test

Comparisons between continuous joint angle kinematic data in the BW condition and each loading condition were performed across the group, including participants of both sexes, to identify global effects of loading. Statistical parametric mapping (SPM) t-tests were used in each instance with *α* = 0.05, as previously described [48, 49]. Kinematic data were estimated as functions using B-splines. A smoothing parameter of 0.01 was used in the fitting procedure. A t-statistic trajectory was created across the gait cycle and assessed in relation to a critical t-statistic, which was determined using a permutation test by randomly shuffling the labels of the curves and recalculating the maximum t-statistic using these new labels. The analysis was done using R (version 3.6.0) and code used can be accessed at https://github.com/ktrounson/WR-running/blob/master/FDA%20t-test.

#### Generalised additive model

Generalised additive models (GAMs) were fit to continuous joint angle data, with separate GAMs for each joint. In each case, data was modelled as a function of the percentage of the stride cycle. Cyclic cubic regression splines were used to generate basis functions for each condition and smoothing was achieved using the restricted maximum likelihood method. Cubic regression splines are more appropriate for functional data that represent repeated cycles of the same event [50]. The number of knots was increased until the maximum deviance explained by the model was reached.

For visualisation of joint kinematic trends and between-run variability on an individual basis, runs from each participant were treated as random effects. The random effects estimates were plotted as a function of condition within each participant. Female participants are labelled F1-F3 and male participants are labelled M1-M11. All GAM code is provided at https://github.com/ktrounson/WR-running/blob/master/GAMs.

#### Bivariate functional principal component analysis

Bivariate functional principal component analysis (*bf*PCA) applied to angle-angle kinematic data allows for the dominant modes of variation to be estimated. *bf*PCA was used to analyse concurrent hip-knee and knee-ankle kinematics using B-spline basis functions [51–53]. The smoothing parameter was selected using a generalised cross validation procedure and was set at 0.1 and 0.18 for the hip-knee and knee-ankle data, respectively. *bf*PCs were derived from the smoothed curves. Each *bf*PC was varimax rotated to assist with interpretation of results. The occurrence and magnitude of angle-angle variability was graphically represented by the first two *bf*PCs on individual plots containing the ensemble mean of curves along with two additional curves representing +/- 2SD of the *bf*PC scores for each *bf*PC. *bf*PCA was performed in R with code available at https://github.com/ktrounson/WR-running/blob/master/bfPCA.

Individual-based 2D plots were generated in which mean *bf*PC scores for each condition were mapped along the first two *bf*PCs for each joint pairing. Positive scores along a dimension indicate that, on average, runs within this condition resembled more closely the characteristics of the ‘+’ curve, while negative scores indicate a closer resemblance to the ‘-’ curve.

## Results

Mean running velocities across participants in each condition are included in Table 1. A significant main effect of condition was evident (*F* = 4.77, *p* = 0.003). Post-hoc analysis showed slower running velocities in the 5% loading condition compared with all other conditions.

**Table 1:** Mean *±* SD running velocities in each condition with post-hoc pairwise comparisons.

Condition	Running velocity (m.s^-1^)	p-value vs. 1%	p-value vs. 3%	p-value vs. 5%
**BW**	4.25 *±* 0.43	1	1	0.017
**1%**	4.25 *±* 0.47		1	0.005
**3%**	4.25 *±* 0.48			0.022
**5%**	4.18 *±* 0.44			

BW, body weight; 1%, 1% of body weight WR loading; 3%, 3% of body weight WR loading; 5%, 5% of body weight WR loading.

### SPM t-test

**Fig 3:**
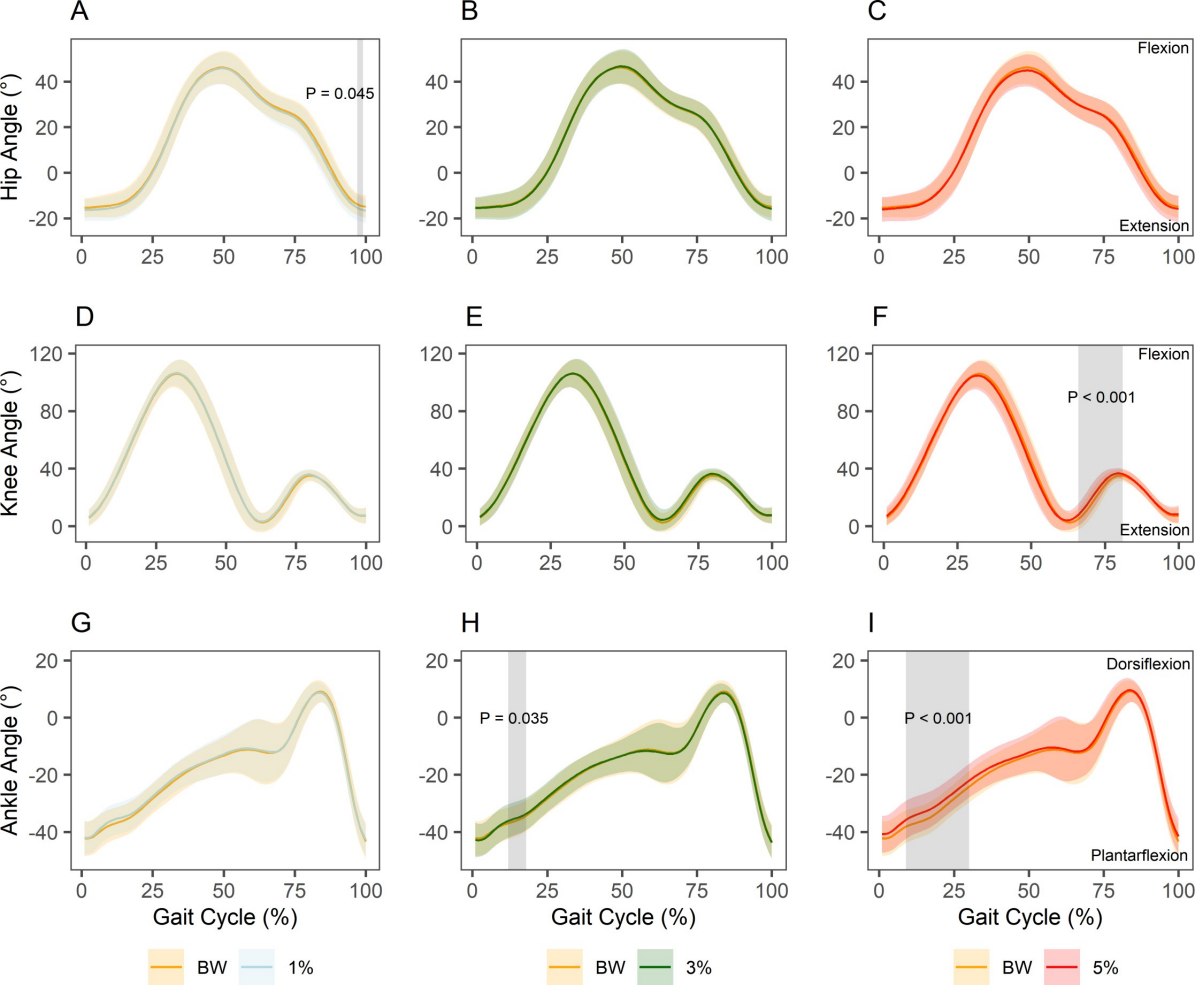
SPM t-test per-joint and per-condition versus BW. (A) Hip joint BW versus 1%. (B) Hip joint BW versus 3%. (C) Hip joint BW versus 5%. (D) Knee joint BW versus 1%. (E) Knee joint BW versus 3%. (F) Knee joint BW versus 5%. (G) Ankle joint BW versus 1%. (H) Ankle joint BW versus 3%. (I) Ankle joint BW versus 5%. Solid lines represent ensemble means and accompanying shaded regions represent ± 1 SD. Grey shaded regions indicate regions of significant difference between curve sets.

Continuous ensemble means per-joint and per-condition with associated standard deviations are presented in Fig 3. Sections of significant difference between the BW condition and each loading condition according to SPM t-tests are indicated. WR loading of 1% of body weight led to greater hip extension at 97–99% of the gait cycle (just prior to toe-off) compared with BW (P = 0.045). Loading of 3% of body weight resulted in less ankle plantarflexion from 12–18% of the gait cycle (during heel recovery) compared with BW (P = 0.035). Loading of 5% of body weight resulted in greater knee flexion from 66–81% of the gait cycle (during weight acceptance) (P < 0.001) and less ankle plantarflexion from 9–30% of the gait cycle (during heel recovery) compared with BW (P < 0.001). Pointwise t-statistics and the maximum critical value for a significance level of 0.05 for each set of curves are provided in S2 Table.

### GAMs

The summary statistics of each joint GAM are shown in Table 2. For BW, the estimate indicates the mean joint angle across the stride cycle. For loading conditions, estimates indicate the difference in mean joint angle across the stride cycle versus BW. Estimated degrees of freedom reflect the number of basis functions used to generate the smooths and therefore a higher number of estimated degrees of freedom suggests more variable data. For loading conditions, estimated degrees of freedom are in addition to those listed for BW.

**Table 2:** Generalised additive model summary statistics per-joint.

		Parametric coefficients				Smooth terms		
Joint	Condition	Estimate	Standard error	t-value	Pr(>|t|)	EDF	F	p-value
**Hip**	**BW (intercept)**	14.16	1.35	10.5	> 0.001	12.96	22666.3	> 0.001
	**1%**	-0.65	0.06	-10.99	> 0.001	3.8	4.94	> 0.001
	**3%**	-0.12	0.06	-2.05	0.04	4.51	3.64	> 0.001
	**5%**	-0.71	0.06	-11.76	> 0.001	7.62	7.12	> 0.001
**Knee**	**BW (intercept)**	43.21	1.15	37.65	> 0.001	8	32137.8	> 0.001
	**1%**	0.55	0.1	5.25	> 0.001	4.05	1.47	0.009
	**3%**	0.71	0.1	6.86	> 0.001	3.54	7.42	> 0.001
	**5%**	0.15	0.1	1.48	0.14	7.29	52.18	> 0.001
**Ankle**	**BW (intercept)**	-18.5	1.06	-17.48	> 0.001	20.85	6141	> 0.001
	**1%**	0.33	0.07	4.95	> 0.001	5.85	2.6	> 0.001
	**3%**	-0.15	0.07	-2.18	0.03	3.91	1.77	> 0.001
	**5%**	1.31	0.07	19.49	> 0.001	5.55	5.06	> 0.001

EDF, estimated degrees of freedom; BW, body weight; 1%, 1% of body weight WR loading; 3%, 3% of body weight WR loading; 5%, 5% of body weight WR loading.

**Fig 4:**
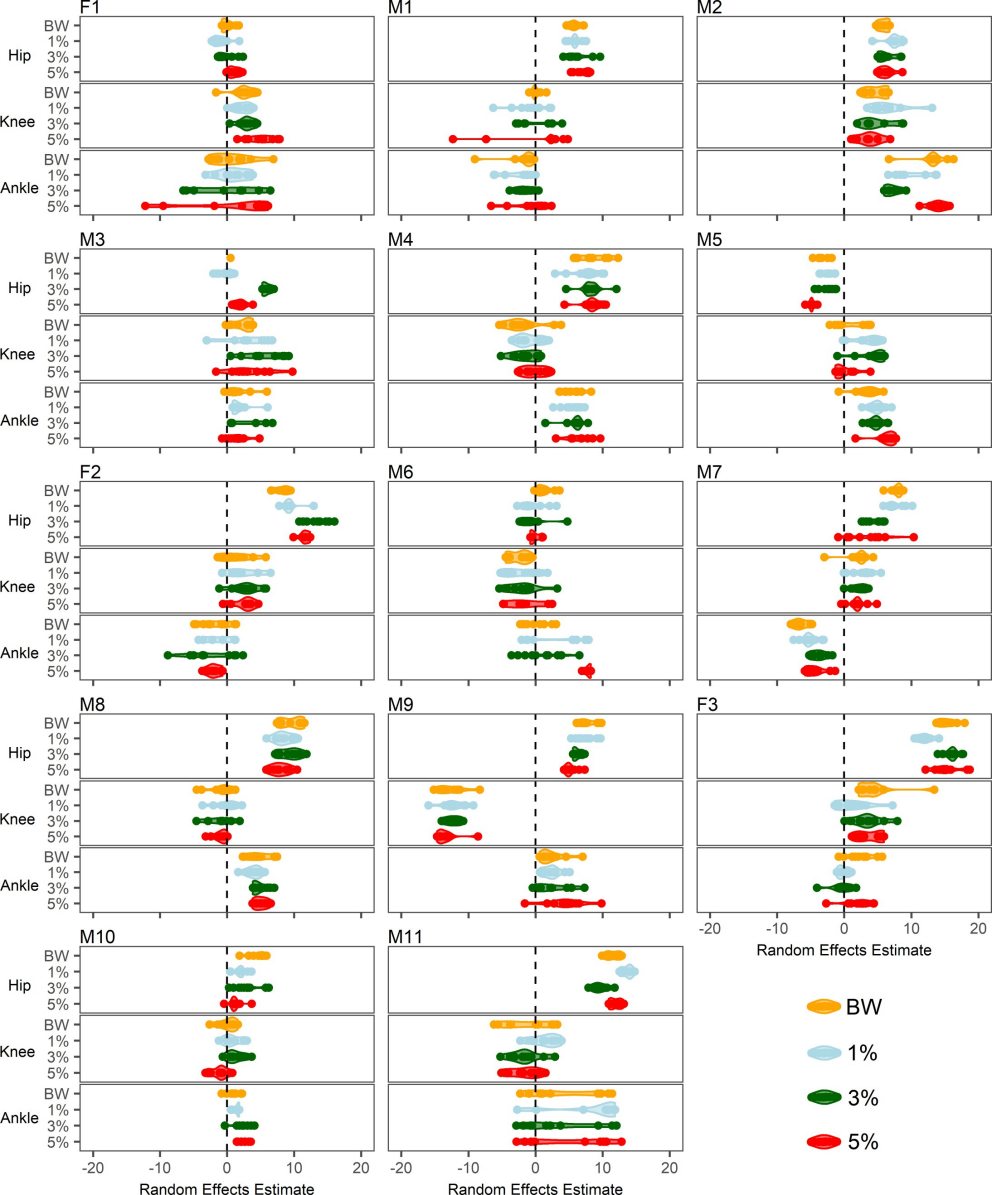
GAM random effects estimates per-run, per-individual. Each major panel relates to a given participant, as denoted by labels. Joints are separated by the three minor panels within each participant plot. Conditions are expressed as categories within each joint and associated colours have been included for clarity. Positive estimates indicate greater hip flexion, knee flexion, and ankle dorsiflexion relative to the group mean. Negative estimates indicate greater hip extension, knee extension, and ankle plantarflexion. Thicker regions of coloured portions reflect a greater concentration of runs with similar random effects estimates.

The GAM random effects estimates per-run are shown in Fig 4. Random effects estimates reflect the prevailing flexion-extension bias throughout the stride cycle relative to the group mean. The distribution of random effects estimates appeared to be more strongly driven by the participant in question than within-participant responses to WR loading. However, individual-level responses of note include instances in which loading increased between-run variability, such as at the ankle in the 5% condition for F1, the knee in the 5% condition for M1, the knee in the 1% condition for M2, the knee in all loading conditions for M3, the hip and ankle in the 3% condition for F2, and the hip in the 5% condition for M7. Conversely, decreased between-run variability was evident at the ankle in the 3% condition for M1, the ankle in the 3% and 5% conditions for M2, the hip in the 5% condition for M5, the ankle in the 5% condition for F2 and M6, and the ankle in the 1% condition for F3. Shifts in the prevailing random effects estimates on the basis of loading appeared evident at the ankle in the 1% and 3% conditions for M2, the hip in the 3% condition for M3 and F2, the ankle in the 5% condition for M6, the hip in the 3% and 5% conditions for M7, the hip in the 1% condition for F3, and the hip in the 1% and 3% conditions for M11.

### *bf*PCA

**Fig 5:**
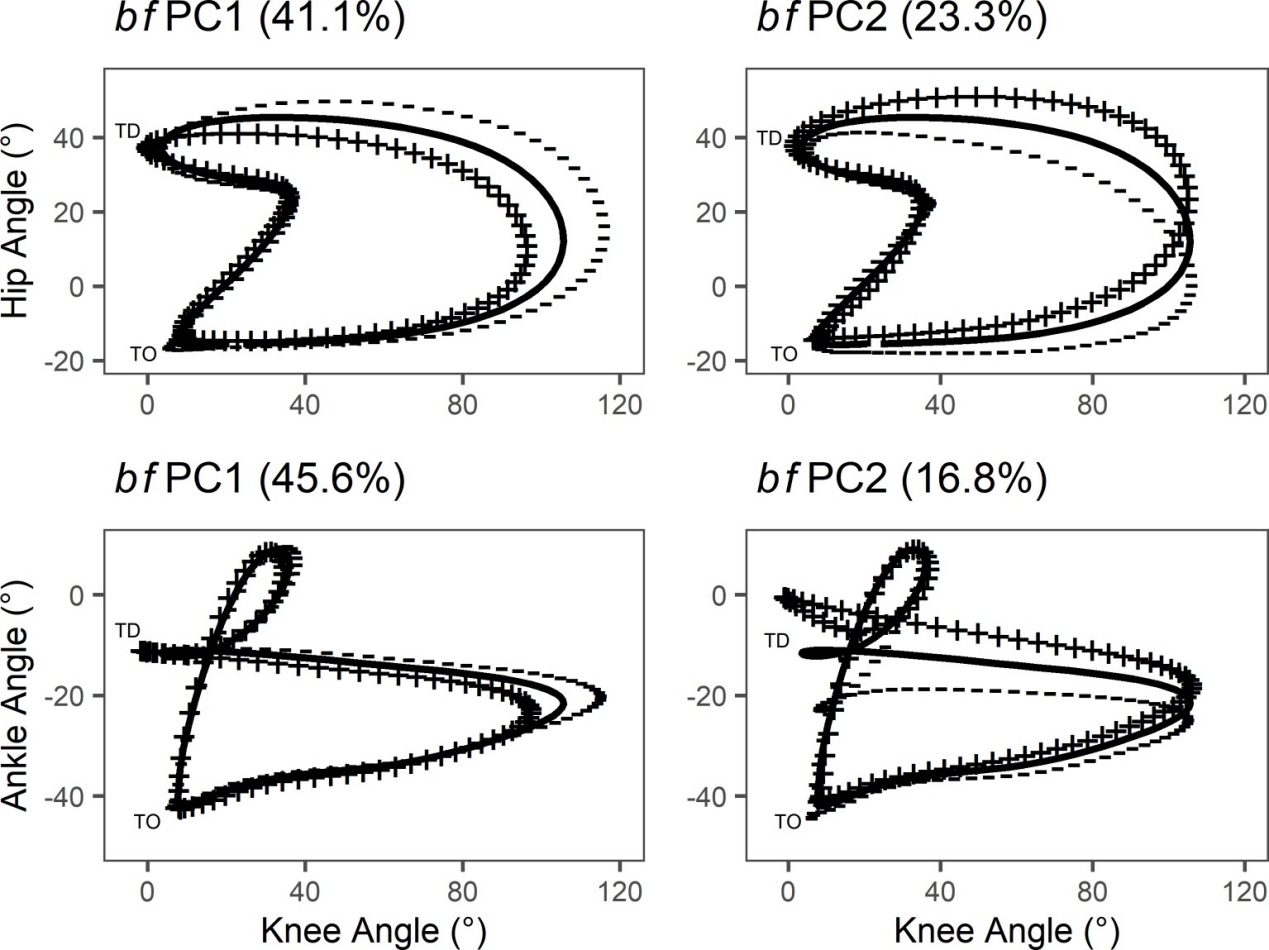
*bf*PCA of hip-knee and knee-ankle joint couples throughout stride cycle. First two bfPCs from hip-knee and knee-ankle *bf*PCA with the percentage of group variability explained. Solid line represents the mean angle-angle curve. ‘+’ line represents positive scorers +2SD from the mean function. ‘-’ line represents negative scorers -2SD from the mean function.

For hip-knee joint coupling, *bf*PC1 explained 41.1% of the variability in the group data (Fig 5). Positive scorers on *bf*PC1 exhibited less knee flexion during the swing phase, while negative scorers exhibited greater knee flexion. *bf*PC2 explained 23.3% of the variability in the group data. Positive scorers on *bf*PC2 exhibited greater hip flexion during the swing phase, while negative scorers exhibited less hip flexion and hip flexion was delayed compared with positive scorers. For knee-ankle joint coupling, *bf*PC1 explained 45.6% of the variability in the group data. Positive scorers exhibited less knee flexion during the swing phase and negative scorers exhibited greater knee flexion. *bf*PC2 explained 16.8% of the variability in the group data. Positive scorers on *bf*PC2 exhibited less ankle plantarflexion, particularly at touchdown, while negative scorers exhibited greater ankle plantarflexion during late swing and touchdown.

**Fig 6:**
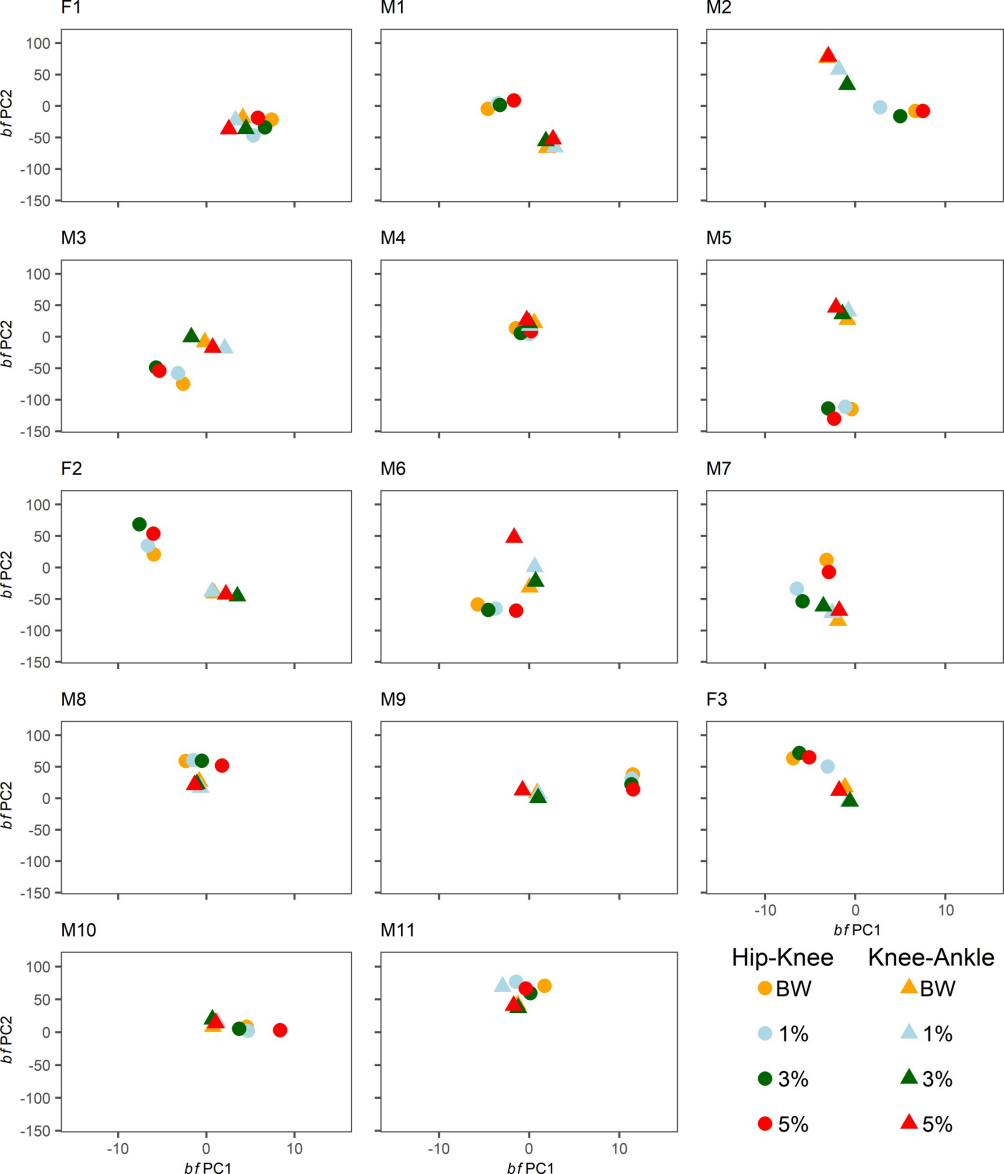
Individual mean hip-knee and knee-ankle *bf*PC1 and *bf*PC2 scores per-condition. Each panel relates to a given participant, as denoted by labels. Mean hip-knee *bf*PC scores across runs within a condition are denoted by circle labels. Mean knee-ankle *bf*PC scores across runs within a condition are denoted by triangle labels. Separate conditions are indicated by distinct colours.

Mean individual *bf*PC scores along both *bf*PCs for hip-knee and knee-ankle joint pairs across runs in each condition are shown in Fig 6. Participants appeared to have mostly distinct joint coupling profiles and there was some impact of WR loading within-individuals. There was generally agreement between observations of condition-based shifts in random effects estimates from GAM analysis and differences in mean *bf*PC scores, including in the knee-ankle joint couple in the 3% condition for M2, the hip-knee joint couple in the 3% condition for M3 and F2, the knee-ankle joint couple in the 5% condition for M6, the hip-knee joint couple in the 3% and 5% conditions for M7, and the hip-knee joint couple in the 1% condition for F3 and M11. Additional condition-based shifts apparent from *bf*PCs included the hip-knee joint couple in the 1% condition in M2, the hip-knee joint couple in the 5% condition for M3, M6, M8, and M10, and the knee-ankle joint couple in the 1% condition for M11. Shifts that were identified from GAM analysis but that appeared to be minimal based on *bf*PC plots included the knee-ankle joint couple in the 1% condition for M3 and the hip-knee joint couple in the 3% condition for M11.

## Discussion

This study examined the effects of lower limb WR loading on coordination tendencies during sub-maximal overground running. Specifically, the study sought to describe the effects of various WR magnitudes (1%, 3%, and 5% of body weight) on lower limb sagittal plane joint kinematics at a group- and individual-level, both in terms of continuous gait cycle kinematics and between-run movement variability. The main findings at a group-level were that 3% and 5% loading decreased ankle plantarflexion during heel recovery, while 5% loading also increased knee flexion during weight acceptance, compared with BW running. In terms of joint coupling, 5% loading brought about the largest changes in coordination at the hip-knee joint pair. At an individual-level, six of the fourteen participants clearly exhibited increased between-run joint angle variability at one or more joints in one or more loading conditions compared with BW.

Running velocity was slower in the 5% condition compared with all other conditions, however, the magnitude of this difference was minimal at just 0.07 m.s^-1^. All participants maintained speeds sufficient to successfully complete all shuttles within the allotted time frames in all conditions.

In terms of kinematics at the group-level, slight decreases in ankle plantarflexion during heel recovery occurred with 3% loading compared with BW running. The 5% loading condition led to more substantial decreases in ankle plantarflexion during heel recovery, as well as increased knee flexion during weight acceptance. The exhibition of greater knee flexion was likely a mechanism to mitigate increased peak ground reaction force arising from the greater system load [54, 55]. Explanations for less plantarflexion during heel recovery are more speculative. One possibility is that participants subconsciously attempted to offset the greater moment of inertia at the thigh by dorsiflexing the ankle to create a mechanical advantage during swing leg recovery [56, 57]. Alternatively, or perhaps in addition, heavier loading likely led to increased co-contraction of muscles around the ankle joint during stance for maintenance of stiffness and stability [55, 58]. Such alterations in motor unit recruitment and temporal sequencing of lower leg muscles may constrain the action of this joint during the subsequent propulsion and swing phase, with the joint returning to a relatively more neutral position more readily [59, 60]. The impact of coordination dynamics should also be considered. Individuals performing novel motor tasks often exhibit freezing of distal biomechanical degrees of freedom to reduce coordinative complexity [61–63]. To the extent that running with an extra 5% of body weight on the lower limbs was perceived as a novel task, there may have been a tendency for participants to return to a more neutral ankle position following toe-off. Given these factors, 5% loading, and to a lesser extent 3% loading, may be excessive as a means of promoting movement variability for some individuals in the first instance. The group-level changes suggest a degree of convergence toward a common adaptation strategy and appear consistent with movement options being limited by task novelty and/or the need to manage high loads. Coaches should take this into consideration if prescribing WR for multiple athletes without individualisation [64].

Despite group-level trends, individual responses varied. The practical utility of WR for inducing movement variability is therefore likely to also be individual-dependent. Coaches should appreciate the range of individual responses and use the present findings as signposts to guide individual WR prescription in the field.

Participants F1, M1, M2, M3, F2, and M7 all exhibited increased between-run variability in mean angles at one or more joints in one or more loading conditions compared with BW. These high variability instances suggest that there was no readily accessible adaptive mode to satisfy the task goal in the presence of WR and instead a period of search and refinement of individuals’ preferred coordinative structures was required [65, 66]. WR in this context therefore provides an opportunity to explore movement system degeneracy. For these individuals, exposure to WR over a training period may facilitate development of movement adaptability and allow running performance to be more readily maintained when perturbations arise in competition [10, 67]. The propensity for individuals to exhibit greater variability at one loading condition over another is largely a function of intrinsic behavioural dynamics, which dictate system tendencies such as attractor state stability and behavioural meta-stability [68, 69].

A definitive reduction in between-run variability was present in participants F2 and M6 at the ankle joint in the 5% loading condition. This may align with the proposed group-level hypothesis of distal joint freezing in this condition. While established literature tends to define freezing degrees of freedom as restricted movement of a joint within-trials, low between-trial variability is also indicative of constrained movement [20, 70]. Among these individuals, the perturbation of 5% loading may have been managed by increasing co-contractions and stiffening muscles of the lower limbs, as occurs in the early stages of skill acquisition [61]. Interestingly, this can be considered an adaptive strategy in itself, particularly since performance of shuttle runs was successfully maintained. This therefore raises the need to clarify the benefit of perturbations that encourage movement variability during training versus those that limit movement variability. Findings from balance beam walking with different perturbation magnitudes demonstrate that learning under conditions in which sacral movement variability is maximised leads to superior learning and subsequent task performance post-training [71]. Substantially increasing the level of perturbation through an error augmenting device decreases movement variability in line with individuals attempting to maintain control of movement, and has suboptimal outcomes for post-training performance [71]. Separately, Chmielewski *et al*. [60] argue that increased co-contractions as a means of adapting to an ACL rupture reflect a suboptimal compensation pattern wherein the capacity to dynamically stabilise the injured knee without compromising knee motion has not yet been developed. Taken together, these findings highlight that large magnitude perturbations may be adapted to by reducing movement variability, however, skill acquisition is not facilitated under these conditions. Reduced movement variability affords fewer opportunities for internal models of limb dynamics to be updated, which may limit the extent to which adaptability is trained [72].

Among high performing field-based athletes, some individuals are likely to already have well developed functional movement adaptability [11, 12]. This is typified by an appropriate mix of movement pattern flexibility and stability, such that coordination can be readily adjusted in response to a perturbation and movement variability levels remain similar to those at baseline [10, 73]. Potential exemplars of this in the present study include participant M10 in all loading conditions and participant M7 in the 3% loading condition.

Participants M4 and M9 exhibited no discernable joint kinematic changes at any loading magnitude. Between-run variability also appeared consistent across loading. For these individuals, loading even up to 5% of body weight may not have required additional exploitation of movement system degeneracy to satisfy the task goal [74]. As part of their intrinsic behavioural dynamics, these individuals likely defer to highly stable movement attractor states in the presence of manageable perturbations [65, 75]. Practically, WR may not be appropriate to challenge running coordination among such individuals, as loading beyond 5% of body weight on the lower limbs presents logistical difficulties due to load placement space limitations.

Lastly, it is interesting to note that participants M2 and M11 appeared to demonstrate multi-stability about the ankle joint. There appeared to be two dominant kinematic modes expressed with apparent condition dependence in M2 but not in M11. Coaches should appreciate that athletes may exhibit multi-stability, wherein two or more patterns of coordination are stable [68].

A limitation of the present study is that only sagittal plane kinematics were considered. Consequential alterations to kinematics may have also occurred in the transverse and frontal planes, or in trunk or upper body segments. In terms of the WR, an exactly equal distribution of load between the anterior and posterior segment surfaces could not always be guaranteed. In these instances, one surface of each segment experienced 50 g more loading, which although minimal, may have impacted ensuing running kinematics. In relation to data processing, it is important to acknowledge that although the initial rise in vertical displacement of the toe marker has previously been used to define toe-off during running actions [46, 47, 76], validation of this detection method against force plate measures under the specific running velocities and floor surface conditions of the present study has not been performed. Lastly, despite the 3 min rest period allowed between running trials, residual after-effects following heavier loading conditions could have briefly impacted on the kinematics observed during lighter conditions [77]. When SPM t-tests were repeated with participants separated on the basis of having completed the 1% condition immediately following the 5% condition as part of their randomisation, it was evident that the group-level differences in hip extension between the 1% condition and BW were driven by these participants (S1 Fig). A larger sample size would provide clarity on this point by enabling direct statistical comparisons between participants who experienced the 1% condition immediately following the 5% condition, and those that did not. If this type of loading contrast was an effectual factor, fidelity could be improved by allowing participants to rest for longer or briefly run without loading in between trials to “re-establish” an unloaded baseline.

Future research should specifically consider the effects of unloaded running immediately following a period of loading to clarify the propensity for acute coordinative changes to be retained following the removal of perturbation. Investigation into the impact of asymmetrical WR loading on coordination would also be worthwhile given the challenge to the movement system that such an intervention would pose. As understanding of the effects of WR loading develops, researchers and/or coaches should consider situating tasks such as loaded running in a representative, field-based environment. WR coupled with the inherent movement variability induced by dynamic constraints and affordances in this environment would present a further, more contextual, challenge to coordination [78].

## Conclusions

Exposure to WR of 5% of body weight increased knee flexion during weight acceptance and decreased ankle plantarflexion during heel recovery at the group-level. This appeared to be due to the high load and novelty of this condition. Among individuals that reflected group-level trends and exhibited decreased between-run variability at one or more joints, 5% loading may be an excessive perturbation, as exploration of alternate movement states is limited. Several participants exhibited increased between-run joint angle variability in one or more loading conditions compared with BW, suggesting exploration and refinement of coordinative structures under these conditions. The loading magnitudes at which these increases were elicited, however, varied between individuals. WR therefore appears to show utility for the purpose of perturbing coordination to encourage movement variability among certain individuals, though the loading magnitudes used should be determined on a case-by-case basis.

## Supporting information

S1 Table List of legs analysed and joint angles unable to be reconstructed for analysis. Joint angle data that could not be reconstructed is highlighted in red.
(DOCX)

S2 Table Pointwise t-statistics and maximum critical values for SPM t-tests. (DOCX)

S1 Fig Hip joint SPM t-test BW versus 1% separated based on condition order. (A) Hip joint BW versus 1% for participants in which 1% condition did not immediately proceed 5% condition. (B) Hip joint BW versus 1% for participants in which 1% condition immediately proceeded 5% condition. Solid lines represent ensemble means and accompanying shaded regions represent ± 1 SD. Grey shaded regions indicate regions of significant difference between curve sets.
(TIF)
