# Supplementary material for: Effects of acute wearable resistance loading on overground running lower body kinematics
Source: PLoS One. 2020 Dec 28;15(12):e0244361. doi: 10.1371/journal.pone.0244361 (PMC7769488; doi:10.1371/journal.pone.0244361)
Supplement: S1 Table — Joint angle data that could not be reconstructed is highlighted in red. (DOCX) [file pone.0244361.s001.docx]

| Participant: F1 | | Joint | | |
| --- | --- | --- | --- | --- |
| Leg analysed: Left | | Hip | Knee | Ankle |
| Loading | Run |  |  |  |
| BW | 1 |  |  |  |
|  | 2 |  |  |  |
|  | 3 |  |  |  |
|  | 4 |  |  |  |
|  | 5 |  |  |  |
|  | 6 |  |  |  |
|  | 7 |  |  |  |
|  | 8 |  |  |  |
|  | 9 |  |  |  |
|  | 10 |  |  |  |
| 1% | 1 |  |  |  |
|  | 2 |  |  |  |
|  | 3 |  |  |  |
|  | 4 |  |  |  |
|  | 5 |  |  |  |
|  | 6 |  |  |  |
|  | 7 |  |  |  |
|  | 8 |  |  |  |
|  | 9 |  |  |  |
|  | 10 |  |  |  |
| 3% | 1 |  |  |  |
|  | 2 |  |  |  |
|  | 3 |  |  |  |
|  | 4 |  |  |  |
|  | 5 |  |  |  |
|  | 6 |  |  |  |
|  | 7 |  |  |  |
|  | 8 |  |  |  |
|  | 9 |  |  |  |
|  | 10 |  |  |  |
| 5% | 1 |  |  |  |
|  | 2 |  |  |  |
|  | 3 |  |  |  |
|  | 4 |  |  |  |
|  | 5 |  |  |  |
|  | 6 |  |  |  |
|  | 7 |  |  |  |
|  | 8 |  |  |  |
|  | 9 |  |  |  |
|  | 10 |  |  |  |
|  | | | | |
| Participant: M1 | | Joint | | |
| Leg analysed: Left | | Hip | Knee | Ankle |
| Loading | Run |  |  |  |
| BW | 1 |  |  |  |
|  | 2 |  |  |  |
|  | 3 |  |  |  |
|  | 4 |  |  |  |
|  | 5 |  |  |  |
|  | 6 |  |  |  |
|  | 7 |  |  |  |
|  | 8 |  |  |  |
|  | 9 |  |  |  |
|  | 10 |  |  |  |
| 1% | 1 |  |  |  |
|  | 2 |  |  |  |
|  | 3 |  |  |  |
|  | 4 |  |  |  |
|  | 5 |  |  |  |
|  | 6 |  |  |  |
|  | 7 |  |  |  |
|  | 8 |  |  |  |
|  | 9 |  |  |  |
|  | 10 |  |  |  |
| 3% | 1 |  |  |  |
|  | 2 |  |  |  |
|  | 3 |  |  |  |
|  | 4 |  |  |  |
|  | 5 |  |  |  |
|  | 6 |  |  |  |
|  | 7 |  |  |  |
|  | 8 |  |  |  |
|  | 9 |  |  |  |
|  | 10 |  |  |  |
| 5% | 1 |  |  |  |
|  | 2 |  |  |  |
|  | 3 |  |  |  |
|  | 4 |  |  |  |
|  | 5 |  |  |  |
|  | 6 |  |  |  |
|  | 7 |  |  |  |
|  | 8 |  |  |  |
|  | 9 |  |  |  |
|  | 10 |  |  |  |
|  | | | | |
| Participant: M2 | | Joint | | |
| Leg analysed: Right | | Hip | Knee | Ankle |
| Loading | Run |  |  |  |
| BW | 1 |  |  |  |
|  | 2 |  |  |  |
|  | 3 |  |  |  |
|  | 4 |  |  |  |
|  | 5 |  |  |  |
|  | 6 |  |  |  |
|  | 7 |  |  |  |
|  | 8 |  |  |  |
|  | 9 |  |  |  |
|  | 10 |  |  |  |
| 1% | 1 |  |  |  |
|  | 2 |  |  |  |
|  | 3 |  |  |  |
|  | 4 |  |  |  |
|  | 5 |  |  |  |
|  | 6 |  |  |  |
|  | 7 |  |  |  |
|  | 8 |  |  |  |
|  | 9 |  |  |  |
|  | 10 |  |  |  |
| 3% | 1 |  |  |  |
|  | 2 |  |  |  |
|  | 3 |  |  |  |
|  | 4 |  |  |  |
|  | 5 |  |  |  |
|  | 6 |  |  |  |
|  | 7 |  |  |  |
|  | 8 |  |  |  |
|  | 9 |  |  |  |
|  | 10 |  |  |  |
| 5% | 1 |  |  |  |
|  | 2 |  |  |  |
|  | 3 |  |  |  |
|  | 4 |  |  |  |
|  | 5 |  |  |  |
|  | 6 |  |  |  |
|  | 7 |  |  |  |
|  | 8 |  |  |  |
|  | 9 |  |  |  |
|  | 10 |  |  |  |
|  | | | | |
| Participant: M3 | | Joint | | |
| Leg analysed: Right | | Hip | Knee | Ankle |
| Loading | Run |  |  |  |
| BW | 1 |  |  |  |
|  | 2 |  |  |  |
|  | 3 |  |  |  |
|  | 4 |  |  |  |
|  | 5 |  |  |  |
|  | 6 |  |  |  |
|  | 7 |  |  |  |
|  | 8 |  |  |  |
|  | 9 |  |  |  |
|  | 10 |  |  |  |
| 1% | 1 |  |  |  |
|  | 2 |  |  |  |
|  | 3 |  |  |  |
|  | 4 |  |  |  |
|  | 5 |  |  |  |
|  | 6 |  |  |  |
|  | 7 |  |  |  |
|  | 8 |  |  |  |
|  | 9 |  |  |  |
|  | 10 |  |  |  |
| 3% | 1 |  |  |  |
|  | 2 |  |  |  |
|  | 3 |  |  |  |
|  | 4 |  |  |  |
|  | 5 |  |  |  |
|  | 6 |  |  |  |
|  | 7 |  |  |  |
|  | 8 |  |  |  |
|  | 9 |  |  |  |
|  | 10 |  |  |  |
| 5% | 1 |  |  |  |
|  | 2 |  |  |  |
|  | 3 |  |  |  |
|  | 4 |  |  |  |
|  | 5 |  |  |  |
|  | 6 |  |  |  |
|  | 7 |  |  |  |
|  | 8 |  |  |  |
|  | 9 |  |  |  |
|  | 10 |  |  |  |
|  | | | | |
| Participant: M4 | | Joint | | |
| Leg analysed: Left | | Hip | Knee | Ankle |
| Loading | Run |  |  |  |
| BW | 1 |  |  |  |
|  | 2 |  |  |  |
|  | 3 |  |  |  |
|  | 4 |  |  |  |
|  | 5 |  |  |  |
|  | 6 |  |  |  |
|  | 7 |  |  |  |
|  | 8 |  |  |  |
|  | 9 |  |  |  |
|  | 10 |  |  |  |
| 1% | 1 |  |  |  |
|  | 2 |  |  |  |
|  | 3 |  |  |  |
|  | 4 |  |  |  |
|  | 5 |  |  |  |
|  | 6 |  |  |  |
|  | 7 |  |  |  |
|  | 8 |  |  |  |
|  | 9 |  |  |  |
|  | 10 |  |  |  |
| 3% | 1 |  |  |  |
|  | 2 |  |  |  |
|  | 3 |  |  |  |
|  | 4 |  |  |  |
|  | 5 |  |  |  |
|  | 6 |  |  |  |
|  | 7 |  |  |  |
|  | 8 |  |  |  |
|  | 9 |  |  |  |
|  | 10 |  |  |  |
| 5% | 1 |  |  |  |
|  | 2 |  |  |  |
|  | 3 |  |  |  |
|  | 4 |  |  |  |
|  | 5 |  |  |  |
|  | 6 |  |  |  |
|  | 7 |  |  |  |
|  | 8 |  |  |  |
|  | 9 |  |  |  |
|  | 10 |  |  |  |
|  | | | | |
| Participant: M5 | | Joint | | |
| Leg analysed: Right | | Hip | Knee | Ankle |
| Loading | Run |  |  |  |
| BW | 1 |  |  |  |
|  | 2 |  |  |  |
|  | 3 |  |  |  |
|  | 4 |  |  |  |
|  | 5 |  |  |  |
|  | 6 |  |  |  |
|  | 7 |  |  |  |
|  | 8 |  |  |  |
|  | 9 |  |  |  |
|  | 10 |  |  |  |
| 1% | 1 |  |  |  |
|  | 2 |  |  |  |
|  | 3 |  |  |  |
|  | 4 |  |  |  |
|  | 5 |  |  |  |
|  | 6 |  |  |  |
|  | 7 |  |  |  |
|  | 8 |  |  |  |
|  | 9 |  |  |  |
|  | 10 |  |  |  |
| 3% | 1 |  |  |  |
|  | 2 |  |  |  |
|  | 3 |  |  |  |
|  | 4 |  |  |  |
|  | 5 |  |  |  |
|  | 6 |  |  |  |
|  | 7 |  |  |  |
|  | 8 |  |  |  |
|  | 9 |  |  |  |
|  | 10 |  |  |  |
| 5% | 1 |  |  |  |
|  | 2 |  |  |  |
|  | 3 |  |  |  |
|  | 4 |  |  |  |
|  | 5 |  |  |  |
|  | 6 |  |  |  |
|  | 7 |  |  |  |
|  | 8 |  |  |  |
|  | 9 |  |  |  |
|  | 10 |  |  |  |
|  | | | | |
| Participant: F2 | | Joint | | |
| Leg analysed: Right | | Hip | Knee | Ankle |
| Loading | Run |  |  |  |
| BW | 1 |  |  |  |
|  | 2 |  |  |  |
|  | 3 |  |  |  |
|  | 4 |  |  |  |
|  | 5 |  |  |  |
|  | 6 |  |  |  |
|  | 7 |  |  |  |
|  | 8 |  |  |  |
|  | 9 |  |  |  |
|  | 10 |  |  |  |
| 1% | 1 |  |  |  |
|  | 2 |  |  |  |
|  | 3 |  |  |  |
|  | 4 |  |  |  |
|  | 5 |  |  |  |
|  | 6 |  |  |  |
|  | 7 |  |  |  |
|  | 8 |  |  |  |
|  | 9 |  |  |  |
|  | 10 |  |  |  |
| 3% | 1 |  |  |  |
|  | 2 |  |  |  |
|  | 3 |  |  |  |
|  | 4 |  |  |  |
|  | 5 |  |  |  |
|  | 6 |  |  |  |
|  | 7 |  |  |  |
|  | 8 |  |  |  |
|  | 9 |  |  |  |
|  | 10 |  |  |  |
| 5% | 1 |  |  |  |
|  | 2 |  |  |  |
|  | 3 |  |  |  |
|  | 4 |  |  |  |
|  | 5 |  |  |  |
|  | 6 |  |  |  |
|  | 7 |  |  |  |
|  | 8 |  |  |  |
|  | 9 |  |  |  |
|  | 10 |  |  |  |
|  | | | | |
| Participant: M6 | | Joint | | |
| Leg analysed: Left | | Hip | Knee | Ankle |
| Loading | Run |  |  |  |
| BW | 1 |  |  |  |
|  | 2 |  |  |  |
|  | 3 |  |  |  |
|  | 4 |  |  |  |
|  | 5 |  |  |  |
|  | 6 |  |  |  |
|  | 7 |  |  |  |
|  | 8 |  |  |  |
|  | 9 |  |  |  |
|  | 10 |  |  |  |
| 1% | 1 |  |  |  |
|  | 2 |  |  |  |
|  | 3 |  |  |  |
|  | 4 |  |  |  |
|  | 5 |  |  |  |
|  | 6 |  |  |  |
|  | 7 |  |  |  |
|  | 8 |  |  |  |
|  | 9 |  |  |  |
|  | 10 |  |  |  |
| 3% | 1 |  |  |  |
|  | 2 |  |  |  |
|  | 3 |  |  |  |
|  | 4 |  |  |  |
|  | 5 |  |  |  |
|  | 6 |  |  |  |
|  | 7 |  |  |  |
|  | 8 |  |  |  |
|  | 9 |  |  |  |
|  | 10 |  |  |  |
| 5% | 1 |  |  |  |
|  | 2 |  |  |  |
|  | 3 |  |  |  |
|  | 4 |  |  |  |
|  | 5 |  |  |  |
|  | 6 |  |  |  |
|  | 7 |  |  |  |
|  | 8 |  |  |  |
|  | 9 |  |  |  |
|  | 10 |  |  |  |
|  | | | | |
| Participant: M7 | | Joint | | |
| Leg analysed: Right | | Hip | Knee | Ankle |
| Loading | Run |  |  |  |
| BW | 1 |  |  |  |
|  | 2 |  |  |  |
|  | 3 |  |  |  |
|  | 4 |  |  |  |
|  | 5 |  |  |  |
|  | 6 |  |  |  |
|  | 7 |  |  |  |
|  | 8 |  |  |  |
|  | 9 |  |  |  |
|  | 10 |  |  |  |
| 1% | 1 |  |  |  |
|  | 2 |  |  |  |
|  | 3 |  |  |  |
|  | 4 |  |  |  |
|  | 5 |  |  |  |
|  | 6 |  |  |  |
|  | 7 |  |  |  |
|  | 8 |  |  |  |
|  | 9 |  |  |  |
|  | 10 |  |  |  |
| 3% | 1 |  |  |  |
|  | 2 |  |  |  |
|  | 3 |  |  |  |
|  | 4 |  |  |  |
|  | 5 |  |  |  |
|  | 6 |  |  |  |
|  | 7 |  |  |  |
|  | 8 |  |  |  |
|  | 9 |  |  |  |
|  | 10 |  |  |  |
| 5% | 1 |  |  |  |
|  | 2 |  |  |  |
|  | 3 |  |  |  |
|  | 4 |  |  |  |
|  | 5 |  |  |  |
|  | 6 |  |  |  |
|  | 7 |  |  |  |
|  | 8 |  |  |  |
|  | 9 |  |  |  |
|  | 10 |  |  |  |
|  | | | | |
| Participant: M8 | | Joint | | |
| Leg analysed: Right | | Hip | Knee | Ankle |
| Loading | Run |  |  |  |
| BW | 1 |  |  |  |
|  | 2 |  |  |  |
|  | 3 |  |  |  |
|  | 4 |  |  |  |
|  | 5 |  |  |  |
|  | 6 |  |  |  |
|  | 7 |  |  |  |
|  | 8 |  |  |  |
|  | 9 |  |  |  |
|  | 10 |  |  |  |
| 1% | 1 |  |  |  |
|  | 2 |  |  |  |
|  | 3 |  |  |  |
|  | 4 |  |  |  |
|  | 5 |  |  |  |
|  | 6 |  |  |  |
|  | 7 |  |  |  |
|  | 8 |  |  |  |
|  | 9 |  |  |  |
|  | 10 |  |  |  |
| 3% | 1 |  |  |  |
|  | 2 |  |  |  |
|  | 3 |  |  |  |
|  | 4 |  |  |  |
|  | 5 |  |  |  |
|  | 6 |  |  |  |
|  | 7 |  |  |  |
|  | 8 |  |  |  |
|  | 9 |  |  |  |
|  | 10 |  |  |  |
| 5% | 1 |  |  |  |
|  | 2 |  |  |  |
|  | 3 |  |  |  |
|  | 4 |  |  |  |
|  | 5 |  |  |  |
|  | 6 |  |  |  |
|  | 7 |  |  |  |
|  | 8 |  |  |  |
|  | 9 |  |  |  |
|  | 10 |  |  |  |
|  | | | | |
| Participant: M9 | | Joint | | |
| Leg analysed: Left | | Hip | Knee | Ankle |
| Loading | Run |  |  |  |
| BW | 1 |  |  |  |
|  | 2 |  |  |  |
|  | 3 |  |  |  |
|  | 4 |  |  |  |
|  | 5 |  |  |  |
|  | 6 |  |  |  |
|  | 7 |  |  |  |
|  | 8 |  |  |  |
|  | 9 |  |  |  |
|  | 10 |  |  |  |
| 1% | 1 |  |  |  |
|  | 2 |  |  |  |
|  | 3 |  |  |  |
|  | 4 |  |  |  |
|  | 5 |  |  |  |
|  | 6 |  |  |  |
|  | 7 |  |  |  |
|  | 8 |  |  |  |
|  | 9 |  |  |  |
|  | 10 |  |  |  |
| 3% | 1 |  |  |  |
|  | 2 |  |  |  |
|  | 3 |  |  |  |
|  | 4 |  |  |  |
|  | 5 |  |  |  |
|  | 6 |  |  |  |
|  | 7 |  |  |  |
|  | 8 |  |  |  |
|  | 9 |  |  |  |
|  | 10 |  |  |  |
| 5% | 1 |  |  |  |
|  | 2 |  |  |  |
|  | 3 |  |  |  |
|  | 4 |  |  |  |
|  | 5 |  |  |  |
|  | 6 |  |  |  |
|  | 7 |  |  |  |
|  | 8 |  |  |  |
|  | 9 |  |  |  |
|  | 10 |  |  |  |
|  | | | | |
| Participant: F3 | | Joint | | |
| Leg analysed: Right | | Hip | Knee | Ankle |
| Loading | Run |  |  |  |
| BW | 1 |  |  |  |
|  | 2 |  |  |  |
|  | 3 |  |  |  |
|  | 4 |  |  |  |
|  | 5 |  |  |  |
|  | 6 |  |  |  |
|  | 7 |  |  |  |
|  | 8 |  |  |  |
|  | 9 |  |  |  |
|  | 10 |  |  |  |
| 1% | 1 |  |  |  |
|  | 2 |  |  |  |
|  | 3 |  |  |  |
|  | 4 |  |  |  |
|  | 5 |  |  |  |
|  | 6 |  |  |  |
|  | 7 |  |  |  |
|  | 8 |  |  |  |
|  | 9 |  |  |  |
|  | 10 |  |  |  |
| 3% | 1 |  |  |  |
|  | 2 |  |  |  |
|  | 3 |  |  |  |
|  | 4 |  |  |  |
|  | 5 |  |  |  |
|  | 6 |  |  |  |
|  | 7 |  |  |  |
|  | 8 |  |  |  |
|  | 9 |  |  |  |
|  | 10 |  |  |  |
| 5% | 1 |  |  |  |
|  | 2 |  |  |  |
|  | 3 |  |  |  |
|  | 4 |  |  |  |
|  | 5 |  |  |  |
|  | 6 |  |  |  |
|  | 7 |  |  |  |
|  | 8 |  |  |  |
|  | 9 |  |  |  |
|  | 10 |  |  |  |
|  | | | | |
| Participant: M10 | | Joint | | |
| Leg analysed: Left | | Hip | Knee | Ankle |
| Loading | Run |  |  |  |
| BW | 1 |  |  |  |
|  | 2 |  |  |  |
|  | 3 |  |  |  |
|  | 4 |  |  |  |
|  | 5 |  |  |  |
|  | 6 |  |  |  |
|  | 7 |  |  |  |
|  | 8 |  |  |  |
|  | 9 |  |  |  |
|  | 10 |  |  |  |
| 1% | 1 |  |  |  |
|  | 2 |  |  |  |
|  | 3 |  |  |  |
|  | 4 |  |  |  |
|  | 5 |  |  |  |
|  | 6 |  |  |  |
|  | 7 |  |  |  |
|  | 8 |  |  |  |
|  | 9 |  |  |  |
|  | 10 |  |  |  |
| 3% | 1 |  |  |  |
|  | 2 |  |  |  |
|  | 3 |  |  |  |
|  | 4 |  |  |  |
|  | 5 |  |  |  |
|  | 6 |  |  |  |
|  | 7 |  |  |  |
|  | 8 |  |  |  |
|  | 9 |  |  |  |
|  | 10 |  |  |  |
| 5% | 1 |  |  |  |
|  | 2 |  |  |  |
|  | 3 |  |  |  |
|  | 4 |  |  |  |
|  | 5 |  |  |  |
|  | 6 |  |  |  |
|  | 7 |  |  |  |
|  | 8 |  |  |  |
|  | 9 |  |  |  |
|  | 10 |  |  |  |
|  | | | | |
| Participant: M11 | | Joint | | |
| Leg analysed: Right | | Hip | Knee | Ankle |
| Loading | Run |  |  |  |
| BW | 1 |  |  |  |
|  | 2 |  |  |  |
|  | 3 |  |  |  |
|  | 4 |  |  |  |
|  | 5 |  |  |  |
|  | 6 |  |  |  |
|  | 7 |  |  |  |
|  | 8 |  |  |  |
|  | 9 |  |  |  |
|  | 10 |  |  |  |
| 1% | 1 |  |  |  |
|  | 2 |  |  |  |
|  | 3 |  |  |  |
|  | 4 |  |  |  |
|  | 5 |  |  |  |
|  | 6 |  |  |  |
|  | 7 |  |  |  |
|  | 8 |  |  |  |
|  | 9 |  |  |  |
|  | 10 |  |  |  |
| 3% | 1 |  |  |  |
|  | 2 |  |  |  |
|  | 3 |  |  |  |
|  | 4 |  |  |  |
|  | 5 |  |  |  |
|  | 6 |  |  |  |
|  | 7 |  |  |  |
|  | 8 |  |  |  |
|  | 9 |  |  |  |
|  | 10 |  |  |  |
| 5% | 1 |  |  |  |
|  | 2 |  |  |  |
|  | 3 |  |  |  |
|  | 4 |  |  |  |
|  | 5 |  |  |  |
|  | 6 |  |  |  |
|  | 7 |  |  |  |
|  | 8 |  |  |  |
|  | 9 |  |  |  |
|  | 10 |  |  |  |

|  |
| --- |

Denotes data that was not successfully reconstructed.
