# Supplementary material for: Effects of acute wearable resistance loading on overground running lower body kinematics
Source: PLoS One. 2020 Dec 28;15(12):e0244361. doi: 10.1371/journal.pone.0244361 (PMC7769488; doi:10.1371/journal.pone.0244361)
Supplement: S2 Table — (DOCX) [file pone.0244361.s002.docx]

|  | **Pointwise t-statistic** | | | | | | | | |
| --- | --- | --- | --- | --- | --- | --- | --- | --- | --- |
|  | **Hip** | | | **Knee** | | | **Ankle** | | |
|  | **BW vs. 1%** | **BW vs. 3%** | **BW vs. 5%** | **BW vs. 1%** | **BW vs. 3%** | **BW vs. 5%** | **BW vs. 1%** | **BW vs. 3%** | **BW vs. 5%** |
| **95% quantile** | **2.65** | **2.83** | **2.84** | **2.85** | **2.87** | **2.83** | **2.64** | **2.83** | **3.04** |
| Gait % |  |  |  |  |  |  |  |  |  |
| 1 | 2.15 | 0.13 | 0.17 | 0.67 | 0.77 | 1.74 | 0.67 | 1.82 | 1.90 |
| 2 | 2.19 | 0.16 | 0.21 | 0.81 | 0.52 | 1.74 | 0.81 | 1.77 | 2.09 |
| 3 | 2.22 | 0.20 | 0.07 | 0.98 | 0.29 | 1.72 | 0.98 | 1.72 | 2.22 |
| 4 | 2.24 | 0.26 | 0.36 | 1.20 | 0.10 | 1.67 | 1.20 | 1.71 | 2.30 |
| 5 | 2.23 | 0.35 | 0.67 | 1.46 | 0.05 | 1.61 | 1.46 | 1.75 | 2.38 |
| 6 | 2.21 | 0.46 | 0.67 | 1.72 | 0.17 | 1.55 | 1.72 | 1.86 | 2.49 |
| 7 | 2.18 | 0.55 | 0.66 | 1.96 | 0.27 | 1.51 | 1.96 | 2.02 | 2.66 |
| 8 | 2.13 | 0.61 | 0.70 | 2.16 | 0.34 | 1.47 | 2.16 | 2.22 | 2.87 |
| 9 | 2.07 | 0.66 | 0.75 | 2.31 | 0.40 | 1.44 | 2.31 | 2.43 | 3.12 |
| 10 | 2.02 | 0.70 | 0.78 | 2.39 | 0.44 | 1.42 | 2.39 | 2.63 | 3.37 |
| 11 | 1.98 | 0.74 | 0.72 | 2.41 | 0.48 | 1.39 | 2.41 | 2.80 | 3.59 |
| 12 | 1.94 | 0.76 | 0.54 | 2.37 | 0.51 | 1.37 | 2.37 | 2.94 | 3.77 |
| 13 | 1.90 | 0.77 | 0.38 | 2.28 | 0.53 | 1.34 | 2.28 | 3.02 | 3.90 |
| 14 | 1.86 | 0.76 | 0.25 | 2.15 | 0.54 | 1.32 | 2.15 | 3.06 | 3.97 |
| 15 | 1.80 | 0.74 | 0.18 | 2.01 | 0.55 | 1.29 | 2.01 | 3.04 | 3.99 |
| 16 | 1.73 | 0.74 | 0.16 | 1.88 | 0.54 | 1.27 | 1.88 | 2.99 | 3.98 |
| 17 | 1.65 | 0.76 | 0.12 | 1.75 | 0.54 | 1.23 | 1.75 | 2.91 | 3.93 |
| 18 | 1.56 | 0.68 | 0.10 | 1.65 | 0.52 | 1.19 | 1.65 | 2.83 | 3.87 |
| 19 | 1.46 | 0.45 | 0.20 | 1.57 | 0.51 | 1.13 | 1.57 | 2.76 | 3.79 |
| 20 | 1.36 | 0.28 | 0.28 | 1.51 | 0.49 | 1.05 | 1.51 | 2.70 | 3.70 |
| 21 | 1.26 | 0.27 | 0.07 | 1.48 | 0.47 | 0.95 | 1.48 | 2.66 | 3.60 |
| 22 | 1.17 | 0.38 | 0.30 | 1.47 | 0.45 | 0.83 | 1.47 | 2.64 | 3.50 |
| 23 | 1.06 | 0.48 | 0.64 | 1.49 | 0.43 | 0.68 | 1.49 | 2.62 | 3.40 |
| 24 | 0.96 | 0.27 | 0.84 | 1.53 | 0.40 | 0.53 | 1.53 | 2.62 | 3.32 |
| 25 | 0.87 | 0.08 | 0.85 | 1.58 | 0.36 | 0.36 | 1.58 | 2.62 | 3.25 |
| 26 | 0.76 | 0.20 | 0.65 | 1.64 | 0.29 | 0.18 | 1.64 | 2.62 | 3.21 |
| 27 | 0.62 | 0.11 | 0.51 | 1.70 | 0.21 | 0.00 | 1.70 | 2.62 | 3.17 |
| 28 | 0.59 | 0.04 | 0.53 | 1.75 | 0.12 | 0.18 | 1.75 | 2.61 | 3.14 |
| 29 | 0.68 | 0.15 | 0.47 | 1.78 | 0.03 | 0.36 | 1.78 | 2.59 | 3.10 |
| 30 | 0.65 | 0.12 | 0.45 | 1.78 | 0.05 | 0.55 | 1.78 | 2.56 | 3.04 |
| 31 | 0.49 | 0.01 | 0.53 | 1.75 | 0.12 | 0.74 | 1.75 | 2.51 | 2.97 |
| 32 | 0.60 | 0.11 | 0.35 | 1.70 | 0.17 | 0.92 | 1.70 | 2.44 | 2.88 |
| 33 | 0.90 | 0.42 | 0.10 | 1.62 | 0.21 | 1.09 | 1.62 | 2.37 | 2.78 |
| 34 | 0.98 | 0.52 | 0.32 | 1.52 | 0.25 | 1.24 | 1.52 | 2.30 | 2.67 |
| 35 | 0.87 | 0.36 | 0.27 | 1.41 | 0.28 | 1.37 | 1.41 | 2.24 | 2.55 |
| 36 | 0.72 | 0.17 | 0.20 | 1.28 | 0.31 | 1.49 | 1.28 | 2.18 | 2.44 |
| 37 | 0.76 | 0.15 | 0.32 | 1.15 | 0.35 | 1.57 | 1.15 | 2.13 | 2.32 |
| 38 | 0.85 | 0.18 | 0.50 | 1.02 | 0.38 | 1.64 | 1.02 | 2.07 | 2.21 |
| 39 | 0.93 | 0.20 | 0.64 | 0.88 | 0.41 | 1.70 | 0.88 | 2.02 | 2.09 |
| 40 | 1.01 | 0.20 | 0.75 | 0.76 | 0.44 | 1.73 | 0.76 | 1.95 | 1.97 |
| 41 | 1.05 | 0.16 | 0.82 | 0.66 | 0.47 | 1.76 | 0.66 | 1.87 | 1.84 |
| 42 | 1.06 | 0.11 | 0.86 | 0.58 | 0.50 | 1.79 | 0.58 | 1.79 | 1.71 |
| 43 | 1.04 | 0.04 | 0.88 | 0.52 | 0.53 | 1.80 | 0.52 | 1.69 | 1.59 |
| 44 | 1.02 | 0.02 | 0.91 | 0.50 | 0.56 | 1.81 | 0.50 | 1.59 | 1.49 |
| 45 | 0.99 | 0.10 | 0.94 | 0.51 | 0.58 | 1.82 | 0.51 | 1.49 | 1.41 |
| 46 | 0.95 | 0.19 | 0.96 | 0.54 | 0.60 | 1.83 | 0.54 | 1.39 | 1.35 |
| 47 | 0.92 | 0.27 | 0.98 | 0.59 | 0.62 | 1.83 | 0.59 | 1.30 | 1.31 |
| 48 | 0.89 | 0.34 | 0.99 | 0.64 | 0.63 | 1.83 | 0.64 | 1.21 | 1.29 |
| 49 | 0.87 | 0.40 | 1.00 | 0.69 | 0.64 | 1.82 | 0.69 | 1.12 | 1.28 |
| 50 | 0.87 | 0.44 | 1.00 | 0.72 | 0.65 | 1.80 | 0.72 | 1.04 | 1.28 |
| 51 | 0.88 | 0.48 | 1.02 | 0.74 | 0.67 | 1.76 | 0.74 | 0.96 | 1.27 |
| 52 | 0.88 | 0.53 | 1.03 | 0.73 | 0.70 | 1.72 | 0.73 | 0.87 | 1.24 |
| 53 | 0.89 | 0.57 | 1.04 | 0.69 | 0.74 | 1.66 | 0.69 | 0.78 | 1.17 |
| 54 | 0.92 | 0.59 | 1.02 | 0.64 | 0.80 | 1.58 | 0.64 | 0.67 | 1.07 |
| 55 | 0.95 | 0.61 | 0.98 | 0.58 | 0.87 | 1.47 | 0.58 | 0.56 | 0.95 |
| 56 | 0.99 | 0.62 | 0.91 | 0.52 | 0.97 | 1.33 | 0.52 | 0.45 | 0.83 |
| 57 | 1.04 | 0.63 | 0.82 | 0.46 | 1.10 | 1.15 | 0.46 | 0.36 | 0.72 |
| 58 | 1.10 | 0.62 | 0.74 | 0.41 | 1.25 | 0.91 | 0.41 | 0.29 | 0.62 |
| 59 | 1.17 | 0.61 | 0.65 | 0.37 | 1.45 | 0.60 | 0.37 | 0.25 | 0.55 |
| 60 | 1.25 | 0.59 | 0.55 | 0.36 | 1.67 | 0.17 | 0.36 | 0.23 | 0.50 |
| 61 | 1.32 | 0.56 | 0.45 | 0.36 | 1.90 | 0.38 | 0.36 | 0.24 | 0.46 |
| 62 | 1.40 | 0.52 | 0.37 | 0.38 | 2.08 | 1.03 | 0.38 | 0.26 | 0.43 |
| 63 | 1.47 | 0.48 | 0.30 | 0.40 | 2.19 | 1.72 | 0.40 | 0.29 | 0.40 |
| 64 | 1.53 | 0.44 | 0.24 | 0.41 | 2.20 | 2.34 | 0.41 | 0.32 | 0.39 |
| 65 | 1.59 | 0.40 | 0.19 | 0.39 | 2.14 | 2.81 | 0.39 | 0.34 | 0.38 |
| 66 | 1.63 | 0.37 | 0.14 | 0.34 | 2.05 | 3.11 | 0.34 | 0.36 | 0.39 |
| 67 | 1.66 | 0.36 | 0.10 | 0.26 | 1.97 | 3.28 | 0.26 | 0.38 | 0.42 |
| 68 | 1.67 | 0.38 | 0.06 | 0.17 | 1.92 | 3.36 | 0.17 | 0.41 | 0.47 |
| 69 | 1.66 | 0.42 | 0.02 | 0.08 | 1.90 | 3.39 | 0.08 | 0.45 | 0.56 |
| 70 | 1.63 | 0.47 | 0.01 | 0.03 | 1.92 | 3.40 | 0.03 | 0.50 | 0.68 |
| 71 | 1.61 | 0.48 | 0.01 | 0.01 | 1.96 | 3.40 | 0.01 | 0.54 | 0.85 |
| 72 | 1.61 | 0.47 | 0.05 | 0.05 | 2.01 | 3.40 | 0.05 | 0.54 | 1.04 |
| 73 | 1.62 | 0.42 | 0.19 | 0.12 | 2.07 | 3.41 | 0.12 | 0.48 | 1.21 |
| 74 | 1.64 | 0.31 | 0.31 | 0.22 | 2.15 | 3.44 | 0.22 | 0.38 | 1.32 |
| 75 | 1.67 | 0.22 | 0.21 | 0.32 | 2.27 | 3.50 | 0.32 | 0.25 | 1.38 |
| 76 | 1.69 | 0.33 | 0.04 | 0.40 | 2.44 | 3.60 | 0.40 | 0.11 | 1.40 |
| 77 | 1.71 | 0.50 | 0.00 | 0.44 | 2.65 | 3.75 | 0.44 | 0.05 | 1.39 |
| 78 | 1.75 | 0.51 | 0.09 | 0.42 | 2.79 | 3.91 | 0.42 | 0.22 | 1.38 |
| 79 | 1.81 | 0.41 | 0.36 | 0.32 | 2.76 | 3.92 | 0.32 | 0.42 | 1.36 |
| 80 | 1.89 | 0.24 | 0.84 | 0.15 | 2.51 | 3.62 | 0.15 | 0.66 | 1.33 |
| 81 | 1.99 | 0.07 | 1.15 | 0.10 | 2.15 | 3.05 | 0.10 | 0.95 | 1.28 |
| 82 | 2.10 | 0.44 | 1.21 | 0.40 | 1.78 | 2.38 | 0.40 | 1.28 | 1.20 |
| 83 | 2.20 | 0.69 | 1.28 | 0.75 | 1.46 | 1.75 | 0.75 | 1.66 | 1.09 |
| 84 | 2.34 | 0.83 | 1.51 | 1.11 | 1.20 | 1.20 | 1.11 | 2.04 | 0.96 |
| 85 | 2.37 | 0.95 | 1.56 | 1.46 | 1.02 | 0.75 | 1.46 | 2.38 | 0.80 |
| 86 | 2.37 | 1.03 | 1.38 | 1.72 | 0.92 | 0.42 | 1.72 | 2.60 | 0.65 |
| 87 | 2.46 | 1.11 | 1.35 | 1.85 | 0.88 | 0.20 | 1.85 | 2.63 | 0.51 |
| 88 | 2.48 | 1.16 | 1.58 | 1.87 | 0.88 | 0.09 | 1.87 | 2.44 | 0.41 |
| 89 | 2.46 | 1.15 | 1.82 | 1.81 | 0.92 | 0.06 | 1.81 | 2.09 | 0.37 |
| 90 | 2.49 | 1.16 | 1.88 | 1.70 | 0.98 | 0.12 | 1.70 | 1.63 | 0.38 |
| 91 | 2.49 | 1.10 | 1.65 | 1.55 | 1.05 | 0.24 | 1.55 | 1.14 | 0.45 |
| 92 | 2.43 | 0.93 | 1.28 | 1.36 | 1.11 | 0.42 | 1.36 | 0.64 | 0.56 |
| 93 | 2.40 | 0.82 | 1.16 | 1.12 | 1.15 | 0.64 | 1.12 | 0.15 | 0.71 |
| 94 | 2.48 | 0.83 | 1.26 | 0.84 | 1.14 | 0.88 | 0.84 | 0.32 | 0.92 |
| 95 | 2.55 | 0.84 | 1.27 | 0.53 | 1.09 | 1.11 | 0.53 | 0.76 | 1.16 |
| 96 | 2.61 | 0.86 | 1.16 | 0.18 | 0.98 | 1.30 | 0.18 | 1.14 | 1.43 |
| 97 | 2.68 | 0.90 | 1.08 | 0.17 | 0.85 | 1.41 | 0.17 | 1.45 | 1.70 |
| 98 | 2.75 | 0.99 | 1.09 | 0.52 | 0.69 | 1.45 | 0.52 | 1.69 | 1.95 |
| 99 | 2.85 | 1.01 | 1.03 | 0.84 | 0.55 | 1.41 | 0.84 | 1.85 | 2.15 |
| 100 | 2.38 | 0.64 | 0.51 | 1.11 | 0.43 | 1.33 | 1.11 | 1.96 | 2.29 |
