# Supplementary figures and images for: Effects of acute wearable resistance loading on overground running lower body kinematics
Source: PLoS One. 2020 Dec 28;15(12):e0244361. doi: 10.1371/journal.pone.0244361 (PMC7769488; doi:10.1371/journal.pone.0244361)

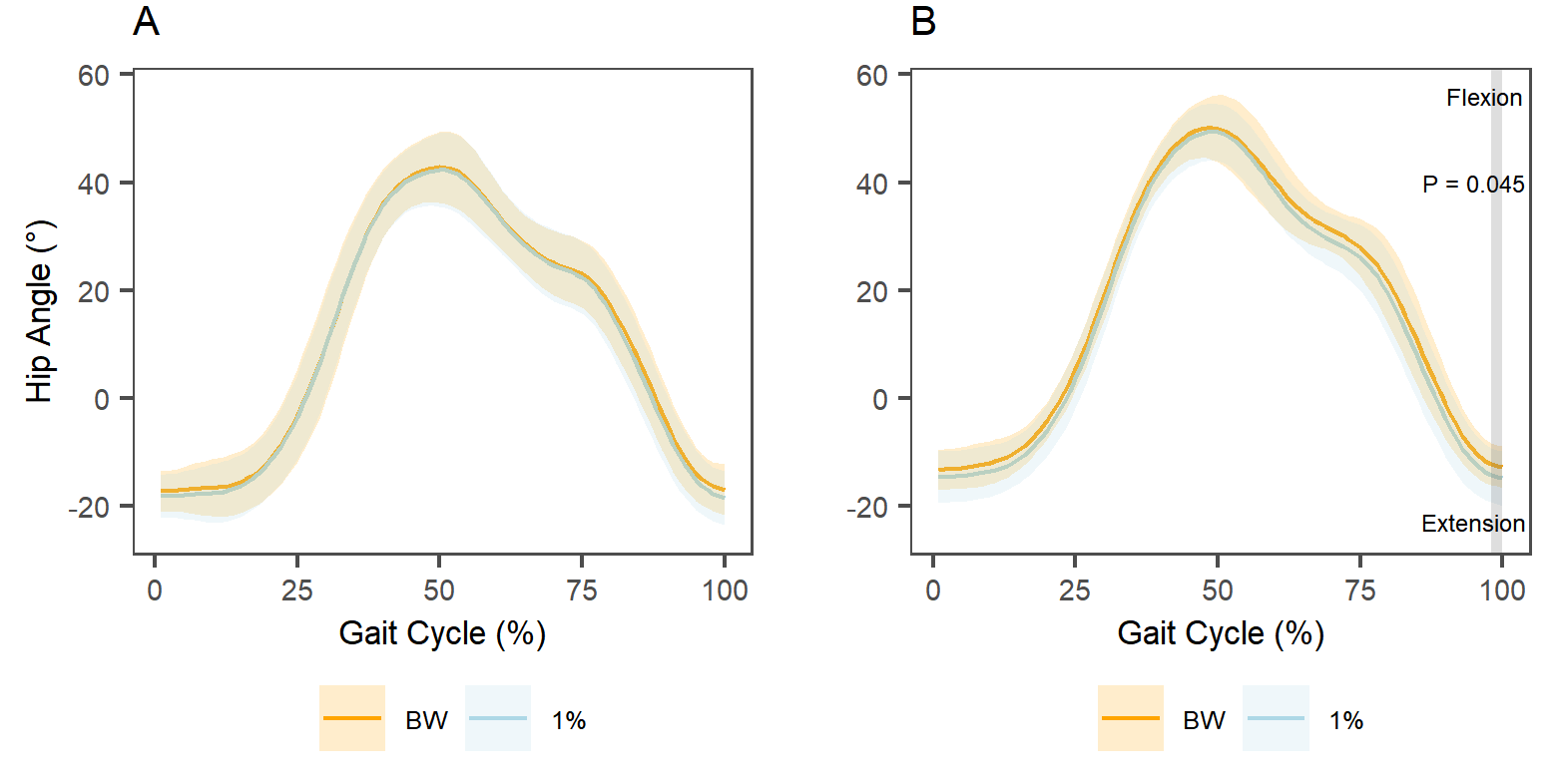

Supplement: S1 Fig — (A) Hip joint BW versus 1% for participants in which 1% condition did not immediately proceed 5% condition. (B) Hip joint BW versus 1% for participants in which 1% condition immediately proceeded 5% condition. Solid lines represent ensemble means and accompanying shaded regions represent ± 1 SD. Grey shaded regions indicate regions of significant difference between curve sets. (TIF) [file pone.0244361.s003.tif]
